# Amino acid sequence based on *Cytochrome b* gene in Kejobong goat and its genetic relationships among several local goats in Asia

**DOI:** 10.14202/vetworld.2018.1196-1202

**Published:** 2018-08-30

**Authors:** Dela Ayu Lestari, Endang Purbowati, Sutopo Sutopo, Edy Kurnianto

**Affiliations:** Department of Animal Science, Faculty of Animal and Agricultural Sciences, Diponegoro University, Tembalang Campus, Semarang 50275, Central Java, Indonesia

**Keywords:** amino acid sequence, *cytochrome b*, Kejobong goat

## Abstract

**Aim:**

This study aimed to analyze the amino acid sequence of *Cytochrome b* (*Cyt b*) gene in Kejobong goat and its genetic relationships with local goats located in Asia.

**Materials and Methods:**

A total of 28 heads of Kejobong goat were purposively sampled. The deoxyribonucleic acid (DNA) was extracted from blood using gSYNC DNA mini kit (Geneaid Biotech Ltd.). *Cyt b* gene was amplified using polymerase chain reaction (PCR) method with CytbCapF and CytbCapR primers. The amplified PCR products were sequenced for further analysis.

**Results:**

There were a total 377 amino acid sequences translated from 1140 base pair (bp) of *Cyt b* gene, 99.20% of it were monomorphic, amino acid alterations were found at site 16^th^, 121^st^, and 231^st^, and Kejobong goat was in the same cluster with Southeast Asian local goats.

**Conclusion:**

Most of the amino acid sequence on *Cyt b* gene in Kejobong goat is monomorphic (99.20%), only a few nucleotide mutations were found that causing amino acid alteration in three sites (0.80%). Kejobong goat has a close genetic relationship to several local goats in Southeast Asian.

## Introduction

There are numerous local goat breeds in Indonesia; one of them is Kejobong goat. By Ministry of Agriculture Republic of Indonesia, Kejobong goat has been lately identified as a new breed of Indonesian local goat with decree No 301/Kpts/SR.120/5/2017 [1]. Kejobong goat can only be found in Purbalingga District, Central Java. It was assumed that Kejobong goat was crossbred of Kacang goat and Etawah Grade goat [2,3]. The most dominant color of Kejobong goat is black (36.84%), black-white (34.21%), black-white (13.16%), black-brown (2.63%), and black-white-brown (13.16%) [4,5]. Kejobong goat has an elongated body shape with firm foot position, both of buck and doe Kejobong have horns with variations in form, udder in doe is large and its shape like a bowl. The features of Kejobong goat are similar to Kacang goat and Etawah Grade goat. Buck Kejobong goat has curve nose like Etawah Grade goat, while doe Kejobong has a straight nose like Kacang goat [6]. Kejobong goat is not only known as a type of meat goat with good productivity but also goat having prolific trait [7]. Research related to the genetic characteristics of Kejobong goat has been done by some researchers [7-10] through nucleotide to analyze genetic diversity, genetic distance, and genetic relationship, but through amino acid sequence analysis was lack. By analyzing amino acid sequence, the variation of evolution rate among three codons forming amino acid can be identified which it will be different in every gene [11]. Moreover, in some studies, using amino acid sequence give better classification results than using nucleotide sequence [12,13].

*Cytochrome b* (*Cyt b*) is one of the genes encoded by mitochondrial deoxyribonucleic acid (mtDNA). The mtDNA sequence has been used extensively in the study of genetic evolution because it is easy to obtain, has a high value in evolution, and generally follows a pattern of inheritance compatible with phylogenetic reconstruction [14]. *Cyt b* is a gene involved in the transport of electrons in the respiratory chain; it can be determined as a target for evolutionary analysis and species identification, particularly useful for comparing species within the same genus or the same family and also can be used to study genetic diversity through mtDNA sequences [15-17]. The uniqueness of *Cyt b* is one of the protein-coding genes that have an eternal part in the species level so that it can be used to categorize breed or to determine genetic relationship [18].

The latest study using *Cyt b* gene for genetic information analysis on goat was done in Iran goat [19], Chinese goat [20,21], Vietnamese goat [22], Turkish goat [23], Indian goat [24], and Indonesian goat [9,10,25-27]. The length of *Cyt b* gene in goat was 1140 bp forming 377 amino acids, preceded with start codon encoded by ATG, end with stop codon encoded by AGA, and located between tRNA^Glu^ and tRNA^Thr^ in mtDNA [28-30]. *Cyt b* is a conserved area or do not change much or do not experience base mutation, so it is more sensitive to use as a genetic marker or barcode to identify purity of species [31-33].

On the basis of the reason, in which there was no information on the sequence of amino acid of *Cyt b* found in local goat, this study was conducted to identify the amino acid sequence of *Cyt b* gene in Kejobong goat and genetic relationships with local goats located in Asia.

## Materials and Methods

### Ethical approval

The materials used in this study have been approved by Animal Ethics Committee for using Animal and Scientific Procedures in Faculty of Animal and Agricultural Sciences, Diponegoro University, Indonesia.

### Materials

A total of 28 heads of Kejobong goat consisting of 15 heads of Kejobong buck (KJ) and 13 heads of Kejobong doe (KB) were purposively sampled at traditional farms in some villages in Kejobong subdistrict, Purbalingga regency, Central Java Province. The goat samples were unrelated genetically based on the information of the owners and local breeding data.

### Methods

Methods of this study consisted of sample collection, DNA extraction, amplification, and sequencing and data analysis.

#### Sample collection

The blood samples about 5 mL of 28 Kejobong goats were collected, and it was taken using 3 cc disposable syringe through *Jugular venous*. Then, it was inserted in Vacutainer’s tubes with anticoagulant (EDTA), stored in a cool box containing ice gel, and transported to the laboratory for analysis.

#### DNA extraction, amplification, and sequencing

**Figure-1:**
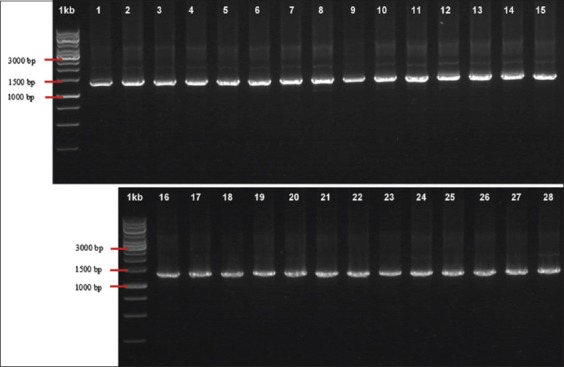
Polymerase chain reaction amplification results of *Cyt b* gene in Kejobong goat.

DNA was carefully extracted from blood based on the manufacturer’s standard protocol using gSYNC DNA mini kit (Geneaid Biotech Ltd, Taiwan.) for sequence analysis *Cyt b* gene in mtDNA. The forward primer CytBCapF (5’-tggaatctaaccatgaccaatg-3’) and reverse primer CytBCapR (5’-ggctattctccttttctggttt-3’) [9] that generated 1261 bp polymerase chain reaction (PCR) product were used to amplify 1140 bp of *Cyt b* gene. The PCR mixture was 50 µL reaction volume containing 25 µL Kappa ready mix, 3 µL DNA template, 1 µL forward primer, 1 µL reverse primer, and 20 µL ddH_2_O. The PCR amplifications were performed using an Infinigen Thermal Cycler by the following program, predenaturation at 94°C for 5 min, followed by 35 cycles, each consisting of 30 s denaturation at 94°C, 45 s primers annealing at 49°C, 90 s elongation at 72°C, then 5 min post-elongation at 72°C for the final stage and were stored at 4°C [10,27]. The PCR products were visualized using 1% Agarose gel. Electrophoresis was run on 100 V condition for 20 min, and the amplification result could be seen in UV light (Figure-1). Sequencing was performed by PT. Genetika Science, Jakarta.

#### Sequence analysis

All mtDNA *Cyt b* sequences were analyzed using Molecular Evolutionary Genetics Analysis 6 program [34] and aligned by ClustalW [35]. The nucleotide sequences then were translated into amino acids form by mitochondrial vertebrate genetic code. Phylogeny tree was constructed based on amino acid sequence by Unweighted Pair Group Method with Arithmetic Mean with p-distance model and 1000 bootstrap replication [36-38] among Kejobong goat and six local goats in Asia as comparator from Genbank [39], those were Laos goat (AB044308); Thailand goat (FJ556557); China goat (EU350133); Korean goat (JX010746); Indian goat (DQ093614); and Japanese goat (D84201).

## Results

### Amino acid sequence and composition

**Figure-2:**
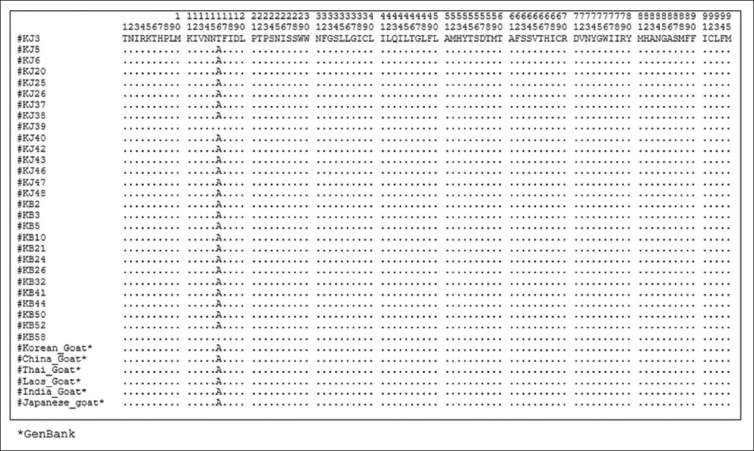
Amino acid sequence from site 1 to 95 of *Cyt b* gene in Kejobong goat.

**Figure-3:**
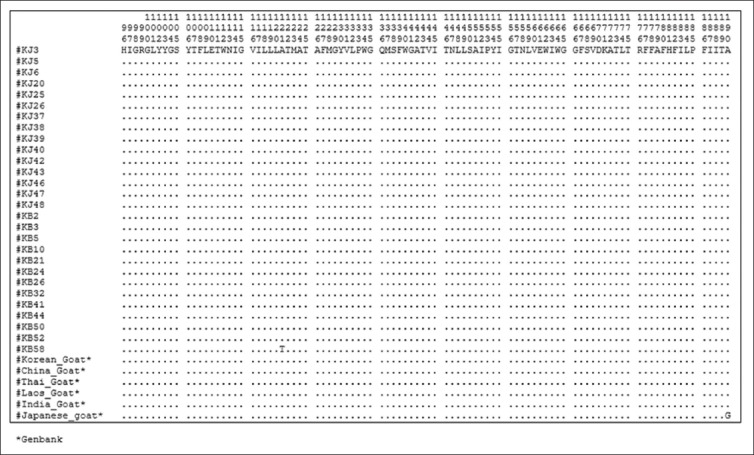
Amino acid sequence from site 96 to 190 of *Cyt b* gene in Kejobong goat.

**Figure-4:**
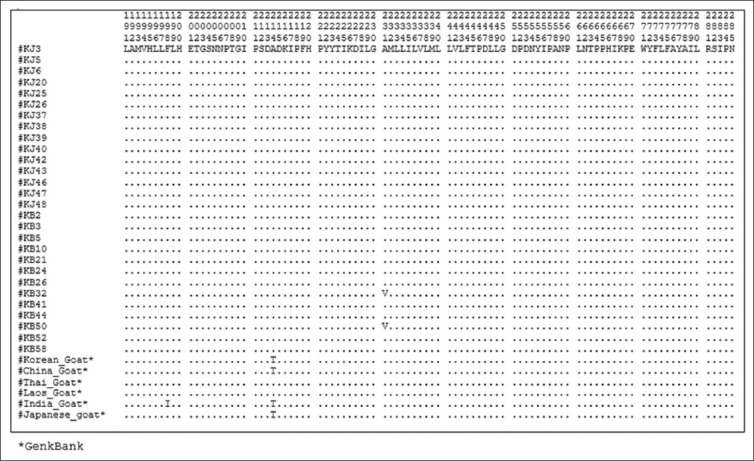
Amino acid sequence from site 191 to 285 of *Cyt b* gene in Kejobong goat.

**Figure-5:**
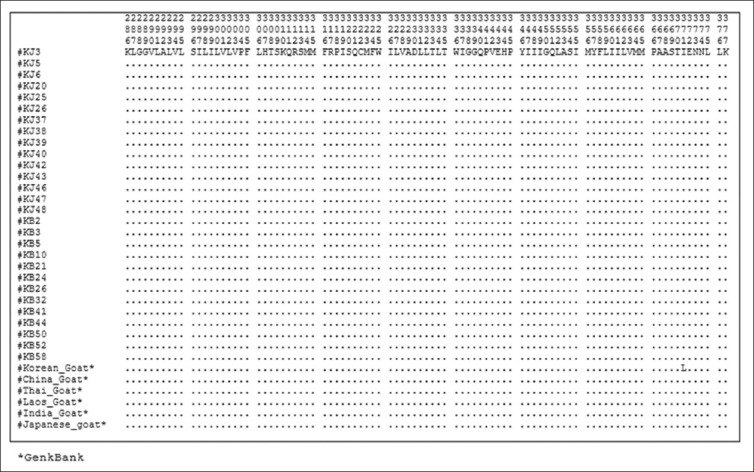
Amino acid sequence from site 286 to 377 of *Cyt b* gene in Kejobong goat.

**Figure-6:**
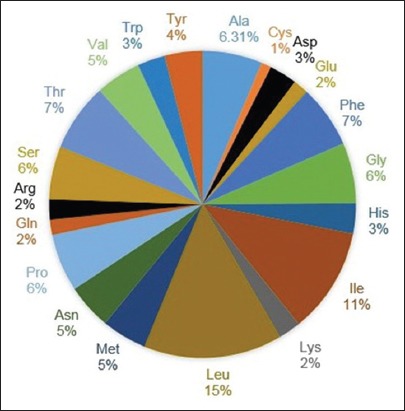
Percentage of amino acid sequence of *Cyt b* gene on Kejobong goat.

A total of 377 amino acid sequences, translated from 1140 (bp) *Cyt b* gene without any deletion/insertion, were obtained from 28 heads of Kejobong goat, (Figures-2-5). The result showed that amino acid sequence in this study consisted of 20 kinds of amino acid with different percentages (Figure-6). It was due to nucleotide polymorphism that formed triplet codons and translated amino acids. Amino acid sequence was dominated by leucine and isoleucine as much as 15% and 11%, respectively, whereas the number of amino acid of cysteine was 1%. Almost all of the amino acid sequences were monomorphic (99.20%), while amino acid alteration was found at site 16^th^, 121^st^, and 231^st^.

### Relationships among Kejobong and several Asian goats

**Figure-7:**
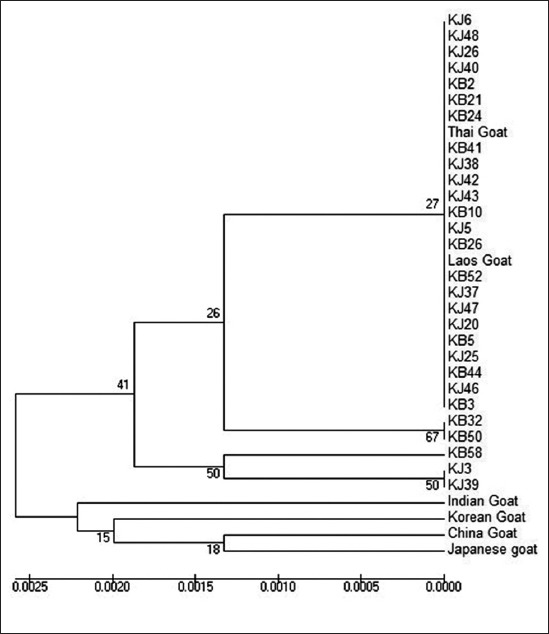
Phylogeny tree of Kejobong goat and several Asian local goats based on amino acid sequence.

The phylogeny tree shows two main clusters which in each cluster there were several subclusters (Figure-7). Almost all of Kejobong goats in this study were in the first cluster together with Thai and Laos goats, but some of them (KB32, KB50, KB58, KJ3, and KJ39) formed separate subcluster with most of the other Kejobong goats. On the other hand, the second cluster was filled by Japanese goat, Korean goat, Chinese goat, and Indian goat.

## Discussion

### Amino acid sequence and composition

**Figure-8:**
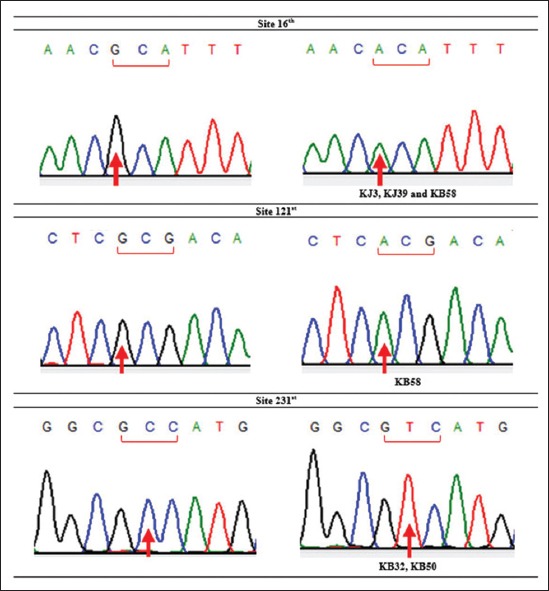
Mutation point of *Cyt b* gene in Kejobong goat.

This result was different from the previous research conducted by Sutopo and Kurnianto [9] that amino acid alteration was found at site 165^th^. The alteration at site 16^th^ occurred because of mutation on nucleotide sequence. Alanine (site 16^th^) encoded by GCA, go through transversions mutation on the first codon from G turned to A in KJ3, KJ39, and KB58, so it became ACA encoding Threonine (Figure-8). The same type of mutation also occurred at site 121^st^ in KB58 from GCG → ACG (Alanine → Threonine), whereas amino acid alteration occurred at site 231^st^ because there was transition mutation on the second codon, from GCC turned GTC (Alanine → Valine) in KB32 and KB50. According to Stansfield *et al*. [40], transition mutation is a point mutation changing a purine nucleotide to another purine or a pyrimidine nucleotide to another pyrimidine, while transversions mutation is a point mutation changing a purine nucleotide to pyrimidine or vice versa. The nucleotide substitution of protein-encoding genes can produce synonymous amino acid (silent substitutions) as well as non-synonymous amino acid. As stated by Nei and Kumar [37], most of synonymous amino acid was found because there was substitution of nucleotides in the third codon, while non-synonymous amino acid was found because of substitution of nucleotides in the first and second codon like in this study. The first and second codon in *Cyt b* gene has a low gamma value (rate of accumulation of substitution). It means that they had a low substitution value or non-variant among individual. However, when there was alteration in the first and second codon, it would cause amino acid alteration [41]. Different from this study, a study performed by Lestari *et al*. [10] investigated genetic diversity of Kejobong goat based on *Cyt b* gene by its nucleotide. It showed three polymorphic sites caused by transition mutation, also found 12 sites that could differentiate clearly between Kejobong goat with *Capra hircus*.

The results of this study were similar to the results of research on Kacang goat and Etawah Grade goat did by Lestari *et al*. [27] in which there was one different amino acid in Kacang goat at site 16^th^ (Alanine → Threonine) from 377 amino acid sequence of *Cyt b* gene. Meanwhile, in Etawah Grade goat, there were two different amino acids and they were at site 16^th^ (Alanine → Threonine) and site 231^st^ (Alanine → Valine). This similarity of amino acid mutation position perhaps related to the assumption that Kejobong goat was a crossbred of Kacang goat and Etawah Grade goat [2,3], and it was confirmed by Lestari *et al*. [42] reporting that by phylogenetic relationship analysis based on D-loop sequence, Kejobong goat was in the same cluster together with Kacang goat and Etawah Grade goat with no clear grouping in each breed in B haplogroup. Meanwhile, as comparator based on other mtDNA genes, Kejobong goat had 11 nucleotide polymorphisms on the basis of 548 bp D-loop sequence that formed 7 haplotypes [8]. Another study conducted by Lestari *et al*. [42], Kejobong goat had 12 nucleotide polymorphisms of 1191 bp D-loop sequence that formed 11 haplotypes with Pi 0.00143±0.00018.

### Genetic relationship among Kejobong goat and several Asian goats

Since there was amino acid alteration in different sites, Kejobong goat was distributed in three subclusters. The cluster based on their amino acid alteration similarity. Kejobong goat in the same cluster with Thai and Laos goats indicated that they were had close genetic distance. This result is in parallel to the report of Pakpahan *et al*. [25] in which local goats in Indonesia have a close genetic distance with several local goats from Southeast Asia as shown in the same cluster position. Other related studies examined the genetic relationship of Kejobong to other breeds indicating that Kejobong goat had a close genetic relationship with *C. hircus* based on nucleotide sequences of *Cyt b* genes [9] and D-loop [8] genes. However, both studies did not give information about the origin of *C. hircus* used as a comparator. Therefore, this result can be used to clarify Kejobong goat phylogenetic status among several Asian local goats.

## Conclusion

This study concluded that most of the amino acid sequence on *Cyt b* gene in Kejobong goat is monomorphic (99.20%), only a few nucleotide mutations were found that causing amino acid alteration in three sites (0.80%). Kejobong goat has a close genetic relationship to several local goats in Southeast Asia.

## Authors’ Contributions

EK planned and conducted the study and corrected the manuscript. DAL carried out the study and wrote the manuscript. EP participated in the drafting manuscript. SS analyzed and interpreted the data analysis result. All authors read and approved the final manuscript.
